# AGORA: Assembly Guided by Optical Restriction Alignment

**DOI:** 10.1186/1471-2105-13-189

**Published:** 2012-08-02

**Authors:** Henry C Lin, Steve Goldstein, Lee Mendelowitz, Shiguo Zhou, Joshua Wetzel, David C Schwartz, Mihai Pop

**Affiliations:** 1 Center for Bioinformatics and Computational Biology, University of Maryland-College Park, College Park, MD, USA; 2 Laboratory for Molecular and Computational Genomics, University of Wisconsin-Madison, Madison, WI, USA; 3 Laboratory of Genetics, University of Wisconsin-Madison, Madison, WI, USA; 4 Department of Chemistry, University of Wisconsin-Madison, Madison, WI, USA; 5 Applied Mathematics and Scientific Computation Program, University of Maryland-College Park, College Park, MD, USA; 6 Department of Computer Science, and Lewis-Sigler Institute for Integrative Genomics, Princeton University, Princeton, NJ, USA

## Abstract

**Background:**

Genome assembly is difficult due to repeated sequences within the genome, which create ambiguities and cause the final assembly to be broken up into many separate sequences (contigs). Long range linking information, such as mate-pairs or mapping data, is necessary to help assembly software resolve repeats, thereby leading to a more complete reconstruction of genomes. Prior work has used optical maps for validating assemblies and scaffolding contigs, after an initial assembly has been produced. However, optical maps have not previously been used within the genome assembly process. Here, we use optical map information within the popular de Bruijn graph assembly paradigm to eliminate paths in the de Bruijn graph which are not consistent with the optical map and help determine the correct reconstruction of the genome.

**Results:**

We developed a new algorithm called AGORA: Assembly Guided by Optical Restriction Alignment. AGORA is the first algorithm to use optical map information directly within the de Bruijn graph framework to help produce an accurate assembly of a genome that is consistent with the optical map information provided. Our simulations on bacterial genomes show that AGORA is effective at producing assemblies closely matching the reference sequences.

Additionally, we show that noise in the optical map can have a strong impact on the final assembly quality for some complex genomes, and we also measure how various characteristics of the starting de Bruijn graph may impact the quality of the final assembly. Lastly, we show that a proper choice of restriction enzyme for the optical map may substantially improve the quality of the final assembly.

**Conclusions:**

Our work shows that optical maps can be used effectively to assemble genomes within the de Bruijn graph assembly framework. Our experiments also provide insights into the characteristics of the mapping data that most affect the performance of our algorithm, indicating the potential benefit of more accurate optical mapping technologies, such as nano-coding.

## Background

Although next generation genome sequencing approaches have improved greatly over the last decade, genome sequencing and assembly still relies primarily on shotgun sequencing [1,2]. Genome assembly, the process of reconstructing the original genome sequence from sequence reads, is made difficult by the fact that the most commonly used sequencing technologies only produce reads between 35 base pairs (bp) and 1 kilo base pairs (kbp) long. Repetitive sequences longer than read lengths lead to ambiguities in the assembly, and additional information from paired-end reads [3] is required to resolve those ambiguities. However, information from paired-end reads is often still insufficient for a comprehensive reconstruction of the original genome sequence [4].

Genome assembly is aided by Optical Mapping--a single molecule system [5-11] for the construction of genome-wide ordered restriction maps through the assembly of (400–500 kbp) genomic DNA, restriction digested and mapped *in situ.* The optical mapping system provides estimates on the locations of restriction-enzyme recognition sequences within a genome. Although optical maps have been used previously to provide a means for scaffolding and validation, in addition to discernment of structural variants [7,11], optical map data is commonly used only *after* a nascent sequence is produced [12] by a genome assembler.

Here, we explore an alternative approach for genome assembly leveraging optical map data within the popular de Bruijn graph assembly paradigm, developing an algorithm we call AGORA: Assembly Guided by Optical Restriction Alignments. We analyze the advantages of utilizing AGORA with optical map information in constructing accurate and comprehensive assemblies. Our algorithm and analysis present the first results showing the benefits of using optical maps *within* the de Bruijn graph assembly paradigm.

Initial simulations show that our algorithm is effective at providing comprehensive assemblies of bacterial genomes, given an optical map with simulated errors and an error-free de Bruijn graph with k-mer size 100. The majority of our assemblies match the original reference sequences very closely. We also measure how the complexity of a genome's repeat structure, reflected in characteristics of the de Bruijn graph, impact AGORA's assembly accuracy. In addition, we investigate how optical mapping error and the choice of restriction enzyme can affect the quality of the final sequence assembly. Moreover, we verify that AGORA works with an experimentally determined optical map from the *Yersinia pestis* KIM genome [13]. Finally, we also explore the applicability of our methods to assembly graphs produced from real sequence reads with errors, and provide a comparison of our results to what can be achieved through the use of mate-pairs (as described in [4]).

### Optical mapping

The Optical Mapping system was first described in 1993 [5,14,15] as a single molecule platform capable of whole genome analysis and as a way to quickly construct physical maps to aid in genome assembly. Optical Mapping produces ordered restriction maps constructed from individual molecules (Rmaps), comprised of an ordered list of restriction fragments identified within each molecule after digestion with a restriction enzyme. The construction of a genome-wide optical map employs assembly techniques akin to those used for sequence assembly [9,10,16], modified to account for error in the Rmaps [9,10,17,18]. The resulting genome-wide optical map produced by this process provides a globally ordered list of restriction fragment sizes across the entire genome.

Previously, algorithms have been developed [13,19-23] to use optical maps to verify and scaffold contigs (partial segments of genome sequence). This scaffolding and validation process is done by first computing for each contig an *in silico* map, which is an ordered restriction map (represented as an ordered list of fragment sizes) constructed computationally by finding all occurrences of the restriction enzyme recognition sequence within each contig. The size and order of the fragments within the *in silico* map are then compared to the sequence of fragments within the optical map of the genome (in a manner analogous to sequence alignment [24]), with the goal of assigning the contig to a single location within the optical map. Recent work by Nagarajan, et al. [12] employs a dynamic programming algorithm to align contigs to an optical map to form a scaffold for the contigs. The validation of contigs is similarly performed by comparing an *in silico* map of each contig with an experimentally determined optical map.

### De Bruijn graph assembly

In this paper, we explore the benefits of using optical maps within the de Bruijn graph genome assembly framework first proposed by Pevzner et al. [25]. A de Bruijn graph is a graph whose nodes correspond to k-mers (sequences of length k) and edges correspond to (k + 1)-mers; an edge may join two nodes if one of the nodes is a prefix of the edge and the other is a suffix. In the context of genome assembly, a node is created for each k-mer in the set of reads and an edge for each (k + 1)-mer. In this formulation, genome assembly is reduced to finding a “Chinese postman path” [26], a path through the de Bruijn graph that visits all edges at least once, which represents the true genome sequence. A full description of this approach is beyond the scope of our paper. Readers interested in more details should refer to [25,27,28].

Practical implementations of the de Bruijn graph assembly paradigm have been used successfully in practice [27-33], and must tackle two major challenges: the presence of sequencing errors, which induce false k-mers in the graph, and the presence of repeats. Due to repeats, the number of Chinese postman paths in the de Bruijn graph can be exponential in the number of nodes and edges [34], making it infeasible to identify the one path that correctly matches the sequence of the genome being assembled. Furthermore, imposing additional constraints on the reconstruction of the genome leads to computationally intractable formulations (see, e.g., [35,36]).

In practice, implementations of this approach forgo the ultimate goal of correctly reconstructing the entire genome sequence and instead attempt to reconstruct a collection of contigs, which generally represent repeat-free sub-paths in the graph. Once these segments have been constructed, additional information from paired-end read data is typically used to resolve repeats and generate scaffolds.

Although paired-end information is generally used only after an initial assembly is produced, recently, Narzisi and Mishra proposed a new algorithm SUTTA [37], which uses paired-end read information within the genome assembly process. The algorithm uses pair-end information to prioritize a greedy branch and bound traversal of the assembly graph according to paired-end constraints, thereby resolving repeats and potentially generating longer contigs. They suggest that optical mapping information could be used in a similar way, but do not provide the details of such an implementation. Here, we design and implement the first algorithm that uses optical mapping data during assembly, employing a framework similar in spirit to the one used by SUTTA, along with several additional improvements. We also use AGORA to explore the effect of the other parameters, such as noise in the optical mapping process, on our ability to effectively reconstruct the sequence of bacterial genomes.

### Overview of AGORA

As outlined above, genome assembly can be effectively formulated as the search for a path within a de Bruijn graph that “spells” the same sequence as the genome being assembled. Optical map information can guide the search for this correct path by eliminating alternate paths that are not consistent with the optical map. To guide the search, an *in silico* map of the sequence corresponding to a partially completed path can be compared to the optical map. If the two maps disagree, we can discard the path as incorrect. As a result, we can quickly prune the set of possible paths, and find a Chinese postman path matching the optical map, which is likely to represent the true reconstruction of the genome. Although imposing map-based constraints on the traversals of the graph leads to computationally intractable problems similar to the Longest Path Problem (a well known problem in computational graph theory, see e.g., [38]) and the Edge Disjoint Paths Problem [39], we show that appropriately chosen heuristics lead to a practical implementation that solves the map-guided assembly problem effectively for bacterial genomes.

A key idea for making our search tractable in practice is the identification of edges within the de Bruijn graph which only match at one location in the genome optical map. These *landmark* edges seed our search, and dramatically reduce the number of paths that need to be investigated. After identifying landmark edges, we then proceed to search for paths connecting pairs of consecutive landmark edges, ensuring that these paths are consistent with the optical map. Although finding a suitable path between consecutive landmark edges may still require exponential time, our experiments on bacterial genomes show that the search process between landmark edges is generally solvable in practice.

To search for paths between landmark edges, we use a refined version of depth first search. As the depth first search proceeds, we check if the *in silico* map of the current path matches the optical map, and if so, we proceed with the depth first search. Otherwise, we backtrack and proceed along a different path until we find a path to the next landmark matching the optical map. With a few additional modifications to the algorithm to improve efficiency (described in the Methods section), AGORA was generally able to find a path in the de Bruijn graph with a sequence and corresponding *in silico* map consistent with the optical map. Although there may be multiple paths in the de Bruijn graph that yield a sequence with an *in silico* map matching the genome optical map, our simulations show that these paths typically yield very similar sequences, differing only in the reconstruction of small complex repeat regions.

## Results and discussion

### Experimental setup

We analyzed the performance of AGORA on 369 sequenced bacterial genomes, using error-free de Bruijn graphs generated from the complete genome sequences as previously described in [34] and optical maps simulated from the sequences. In addition, we also tested AGORA on a published optical map of the *Y. pestis* KIM genome [13]. Note that the error-free de Bruijn graph of a genome sequence of order k is identical to the de Bruijn graph constructed from a collection of error-free sequence reads where every k-mer in the genome is covered by at least one read. The de Bruijn graph of each sequence was simplified by replacing unipaths (a path in which all of the nodes have in-degree = out-degree = 1 [28,32]) with a single edge representing the longer sequence, along with other de Bruijn graph simplifications, which preserve all the information relevant for genome reconstruction from the original de Bruijn graph (see [34] for further details on the simplification procedures). Moreover, we collapsed parallel edges with greater than 99% sequence similarity, as long as the difference in the sequences did not create or remove any restriction sites (see Methods for more details).

To simulate optical maps from a genome sequence, we first compute an *in silico* map of the sequence and then perturb the fragments within this map by sampling from an error distribution. We modeled three different error levels -- *highmedium* and *low* --- and simulated one optical map from each of these distributions to measure the effect of optical mapping error on assembly quality. Although the error simulation is a simple process which may not capture the full characteristics of experimentally generated optical maps (see Methods for details), the results nonetheless show the impact of noise on the final assembly quality. The high error setting has characteristics matching the maximum fragment sizing error and maximum size of small fragments lost observed in the experimental *Y. pestis* KIM optical map, while the low error setting corresponds to what might be achievable with the new nano-coding technology [40]. The low error setting can noticeably improve the performance of our algorithm since it does not remove small fragments from the optical map. A more precise description of the three levels of noise used in our simulations can be found in the Methods section.

After running AGORA, we used four different metrics to measure the quality of the final path through the de Bruijn graph and the sequence associated with it. (See Methods for detailed descriptions of these measures.) Our first metric, *sequence correctness*, roughly corresponds to the percentage of the final sequence that was assembled in the correct order. Our second metric, *edge correctness*, is the number of graph edges placed in the correct order divided by the total number of edges in the de Bruijn graph. The last two metrics measuring the final *N50 size* and *number of contigs* produced by our algorithm are computed after breaking the reconstructed sequence (from the path found by AGORA) wherever an error occurs, and treating sequence segments between errors as independent contigs produced by our assembler. This approach is consistent with the one used by Salzberg et al. [41] in the context of assembly evaluation. These final contig statistics are then compared against the original N50 size and number of contigs that would arise if one were to treat each edge in the starting de Bruijn graph as a separate contig.

### Assembly of bacterial genomes with simulated optical maps

We start by measuring the performance of AGORA on assembling 369 bacterial genomes, providing as input their simplified de Bruijn graphs of order k = 100, which are equivalent to the graphs that can be obtained in an error-free sequencing experiment generating reads longer than 100 bp, covering each k-mer of length 100 in the genome. For each genome, we computationally generated BamHI (recognition sequence G^GATCC) *in silico* maps for simulating optical maps. Statistics summarizing the number of restriction sites in the *in silico* map of each genome and the characteristics of the de Bruijn graphs of the genomes are shown in Table 1.

**Table 1:** Statistics of the de Bruijn graphs and optical maps used in our simulations

	**Min**	**Median**	**Mean**	**Max**
Nodes	1	35	63.63	1,023
Edges	3	110	324.4	14,251
N50 Size (kbp)	14	212.1	419.2	3,587
Genome Length (Mbp)	0.34	2.91	3.2	9.14
Restriction Sites	6	334	491.7	9,668

The de Bruijn graphs for 369 bacterial genomes were generated with k-mer size 100 from the known sequences from [34] (without errors and without bubble collapsing), and the N50 size was computed for each genome, treating each edge in the de Bruijn graph as a separate contig. The row “Restriction Sites” refers to the number of cuts within the genome when using the restriction enzyme BamHI.

Table 1 provides some indication of the complexity of the genomes in our test data set, as measured by their corresponding de Bruijn graphs. The number of nodes in each de Bruijn graph roughly represents the number of distinct repeat sequences longer than 100 bp occurring in the genome, while the number of edges roughly represents the number of times those repeated sequences occur in the genome. Genomes with more nodes and edges in the de Bruijn graph are generally more difficult to assemble, since they contain more repeat sequences.

**Figure 1:**
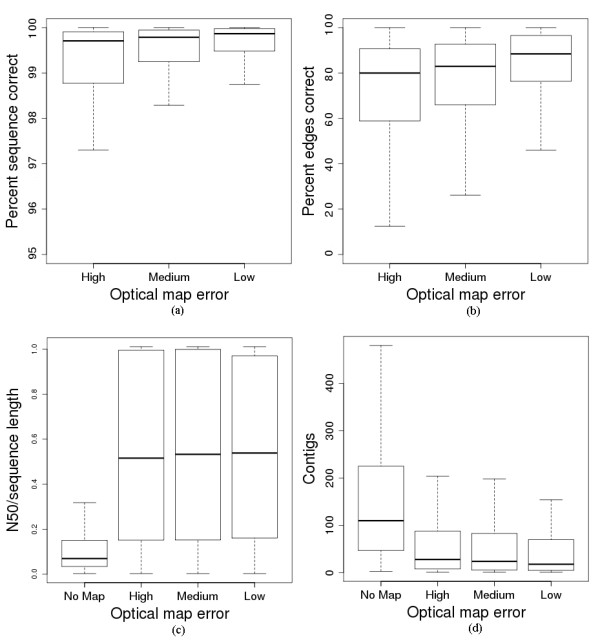
**Assessment of the quality of bacterial genome assemblies.** Measurements of the quality of assemblies produced by our algorithm on 369 bacterial genomes under three different optical map error rates. In each boxplot, we extend the whiskers beyond the upper and lower quartiles for 1.5 times the interquartile range, and omit outliers beyond the whiskers. (**a**) Sequence correctness of the assemblies, measuring the percent of the genome that was correctly assembled. (76, 72, and 65 outliers are not shown the high, medium, and low error bars, respectively.) Over ¾ of the genomes are assembled with greater than 98% sequence correctness, even in the high error setting. (**b**) Percent of edges assembled in the correct order by our algorithm on the 369 genomes, over three error rates. The percent of edges correct is generally lower than the sequence correctness percentages, but the difference is mostly due to short edges misplaced by the algorithm. (26, 33, and 37 outliers are not shown in the high, medium, and low error settings, respectively.) (**c**) N50 size of the final contigs produced by our algorithm, after breaking genomic segments at assembly errors, normalized by genome size. (54 outliers were omitted from the first bar, measuring assembly without a map.) (**d**) Number of contigs that would be produced with no optical map (and only the de Bruijn graph), and with optical maps simulated with three different levels of noise. (We omit 42, 53, 50, and 45 outliers in the no map, high error, medium error, and low error settings, respectively.) We see a substantial improvement in both the final number of contigs and the final N50 size, when given an optical map with any one of the three error rates.

To determine how well we could assemble the 369 bacterial genomes with the help of optical maps, we ran our algorithm on each de Bruijn graph and optical maps simulated with the three different error settings described above. We then measured the quality of our assemblies based on sequence correctness, edge correctness, N50 size, and number of contigs. The results are aggregated in Figure 1, and per-genome information is provided in Additional file 1.

As we can see in Figure 1a, AGORA assembles over ¾ of all genomes with greater than 98% sequence correctness for all three error settings. The mean sequence correctness in the high, medium, and low error settings were 89.2%, 91.9%, and 95.9%, respectively. The means were lower than the median sequence correctness values due to a few very complex genomes for which the algorithm could only assemble a small fraction of the genome, producing outliers which are not shown in the Figure 1. When measuring the number of edges assembled in the correct order as shown in Figure 1b, the edge correctness percentages are lower than the sequence correctness percentages, primarily because edges with short sequences (typically under 1 kbp in length) may be misplaced by our algorithm due to a lack of restriction sites. Although AGORA may misplace 10%-20% of the de Bruijn graph edges, these edges typically contribute to less than 2% of the genome assembled, as indicated by the sequence correctness boxplot shown in Figure 1a.

In Figure 1c and 1d, we plot statistics on the final N50 size and number of contigs that would result if we were to break the final path produced by AGORA wherever a mistake is made, and compare these values with the initial quality of the assembly before using mapping data. We can see in the figures that our algorithm substantially improves the N50 size and number of contigs, even after errors are accounted for. When measuring the overall improvement in N50 size we found that, in the median case, the N50 size increased by a factor of between 3.61 and 4.09, while the mean improvement was between 5.44 and 5.77, depending on the level of mapping error simulated. Similarly, the number of contigs decreased by a factor of between 3.48 and 5.15 in the median case, and the mean improvement was between 6.67 and 10.74. In addition, we found that we had assembled 43, 52, and 69 genomes perfectly into a single contig representing the entire genome sequence in the high, medium, and low settings, respectively.

AGORA finished in under one minute for ¾ of instances, while the longest runtime was around 20 minutes. Note that we forced the algorithm to skip to the next landmark if no path could be found within one minute (see Methods for more details), since our tests required running the algorithm more than 1,000 times. We do not expect this time limitation to significantly affect the median and quartile statistics, as only 18.1% percent of genomes had any regions skipped. Those genomes were generally complex genomes with assembly quality in the lowest quartile, and additional running time did not improve their assembly quality significantly.

**Figure 2:**
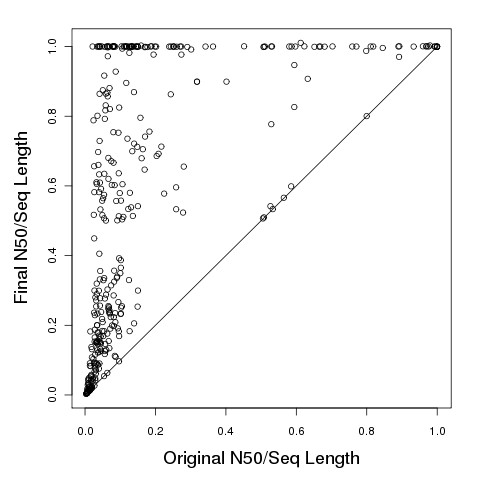
**Improvement in normalized N50 size after assembly.** For each of our 369 bacterial genomes, we plot the initial normalized N50 size (x axis) relative to the normalized N50 size after assembly (y axis) when provided a simulated optical map with the medium error rate, as described in the Methods section. The N50 sizes are normalized by dividing by the genome length. Most genomes exhibit substantial improvement in the normalized N50 size with the exception of complex genomes (with low initial normalized N50 size), and some simple genomes (with initial N50 size already close to the entire genome size).

In addition to the aggregate statistics shown in Figure 1, we also individually compared the original and final N50 sizes produced by our algorithm for each genome using the medium error setting. In Figure 2, we plot a point for each genome at its original and final N50 size, after normalizing by genome length. We see that most genomes have substantial improvement in N50 size, with some genomes even having normalized N50 size starting below 20% and improving to nearly 100% after assembly. Some genomes with normalized N50 size starting below 20% did not improve much, mostly because the corresponding graphs contained many short edges without any restriction sites, making it difficult to place those edges on the optical map and rule out incorrect paths in the de Bruijn graph.

As the normalized N50 size did not seem to predict very well how accurately we could assemble the final genome, we performed further statistical analyses to test whether other factors were more correlated with the final assembly quality. We computed Spearman’s rank correlation coefficient between sequence correctness and various de Bruijn graph characteristics. We found that the sequence correctness obtained by AGORA had the highest correlation with the average edge length of the de Bruijn graph among all the characteristics we measured. The correlations are: genome size (−0.04), normalized N50 size (0.61), N50 size (0.69), number of edges (−0.75), average number of restriction sites per edge (0.76), and average edge length (0.83).

It is not surprising that average edge length, average number of restriction sites per edge, and N50 size have a very strong correlation with sequence correctness as this implies the corresponding de Bruijn graph has long edges which are likely to contain multiple restriction sites. These long edges are easier to place unambiguously along the map and can be used to rule out incorrect de Bruijn graph paths very effectively.

It is important to note that the average edge length and number of edges are strongly anti-correlated (−0.90 Spearman’s coefficient) due to the fact that the genome lengths in our dataset are within a fairly narrow range of 1–5 Mbp (mega base pairs). Given our data, we cannot fully distinguish between the impact of long edges versus fewer edges (lower complexity) on our ability to reconstruct a genome. Genome length also has very low correlation with the sequence correctness of the assembly, but more testing needs to be done on larger and more complex genomes in order to better determine the factors that most influence the quality of genome assembly.

**Figure 3:**
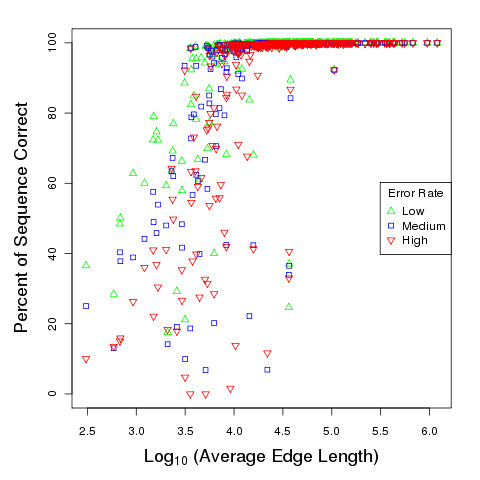
**Impact of average edge length of de Bruijn graph on sequence correctness of assembly.** A plot showing the sequence correctness of 369 bacterial genome assemblies versus the average edge length of their starting de Bruijn graphs, under three different optical map error rates. Genomes with average edge length greater than 10 kbp are generally assembled with near perfect correctness over all three error rates, while the results are mixed for genomes with shorter average edge lengths. For genomes with average edge length below 10 kbp, correctness may improve by as much as 40% when moving from the high error to low error setting, highlighting the potential benefits of more accurate mapping technologies.

We also directly plotted sequence correctness versus the average edge length in each de Bruijn graph over all three error rates (Figure 3) and observed that sequence correctness generally increases with average edge length. Almost all genomes with average edge length greater than 10 kbp can be assembled with accuracy over 98%, even when relying on maps with the highest error rate, while we have mixed results for genomes with shorter average edge length. Also, note the impact of error level in the optical map on the assembly accuracy is less than 2% for genomes with average edge length greater than 10 kbp (which includes 79.9% of our genomes), but has a greater impact on graphs with shorter edges, where the final sequence correctness differs by as much as 40 percentage points between the high and low error settings. Additionally, we find that most genomes with a starting N50 size larger than approximately 50 kbp also yield map-guided assemblies with greater than 98% accuracy. (See Additional file 2.)

### Assembly of *Y. pestis* KIM with previously published optical map

We also evaluated the performance of AGORA on the assembly of the genome of *Y. pestis* KIM (NCBI accession NC_004088) with a PvuII (recognition sequence CAG^CTG) optical map experimentally determined in [13]. In addition to the experimental optical map, we also ran AGORA on optical maps simulated for PvuII with the same three levels of noise mentioned previously. Since *Y. pestis* is a complex genome containing many repeats (primarily IS elements), we provided AGORA with a de Bruijn graph produced with a larger k-mer size of 500 in the initial experiment. Subsequent experiments for k-mer size 100 yield lower quality assemblies, as described in the next section. AGORA took under 5 minutes to find a path matching the genome optical map for each error rate (without having to skip any regions between landmarks due to the 1 minute timeout). The resulting reconstruction of the genome matched the correct sequence with accuracy between 86.74% and 99.13%, depending on the error rate used (see Table 2).

**Table 2:** **Statistics on the assembly of*Y. Pestis*KIM with optical maps of different error rates**

	**Sequence Correct**	**Edges Correct**	**Landmarks**	**Final Contigs**	**N50 Size**
Low Error	99.13%	192/199	64	12	1,190,834
Med Error	97.57%	188/199	38	20	905,369
High Error	90.52%	169/199	25	81	776,452
Map from [13]	86.74%	149/199	26	80	405,321

A summary of the results of our algorithm on assembling *Y. Pestis* KIM, when given a de Bruijn graph with k-mer size 500, and a simulated optimal or experimental optical map from [13].

Optical mapping information substantially improves the initial N50 size of 62,865 bp (computed from the de Bruijn graph of this genome with k-mer size 500) by a factor of between 6.4 and 18.9 depending on the quality of the optical map. The number of contigs is correspondingly reduced by a factor of between 2.45 and 16.6. While the maximum fragment sizing error and maximum size of small fragments lost in the high error optical map simulation match the values observed the experimentally produced optical map [13] (10% and 2 kbp sizing error and loss of fragments smaller than 2 kbp), AGORA generates a slightly worse assembly when guided by the experimental map. This indicates that the simple heuristic procedure we used to simulate noise may not adequately match the precise characteristics of the noise seen in experimentally determined optical maps (see Methods for more details). Nonetheless, our results still show the potential impact of noise in the optical on the quality of the final assembly.

**Figure 4:**
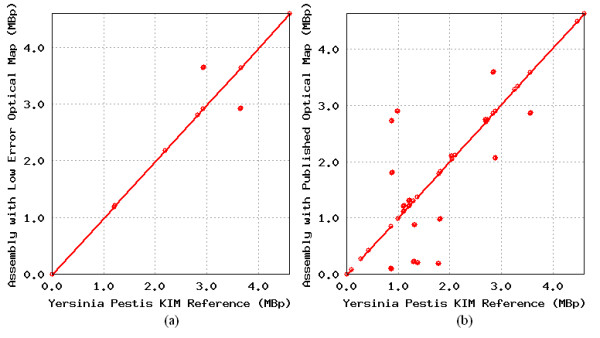
**Mummerplot comparison of assemblies produced with low error and experimental optical map.** Two dot plots generated by Mummerplot comparing the known genome sequence of *Y. pestis* KIM to the sequence assembly produced by our algorithm, when given an optical map with low error added (**a**), and when using the experimental optical map from [13] (**b**).

To further assess the quality of the assemblies, we used Mummer [42] to compare the sequences produced by our algorithm to the known genome sequence. Figure 4a illustrates our previous analysis showing that the sequence generated by AGORA matches the original sequence with greater than 99% accuracy, when given an optical map with low noise. The line along the diagonal indicates sequence that correctly matches the true genome, while only 7 errors can be seen at the locations marked by small circles, which occur due to short misplaced edges. In Figure 4b, we see that there are more errors in the assembly built using the experimental optical map. The gaps in the line along the diagonal in Figure 4b indicate roughly 13% of the genome is not correctly assembled by our algorithm. The longest regions of incorrectly assembled sequence occur in portions of the genome where there are few restriction sites.

In these regions, AGORA picks a single path among several possible paths which may match the optical map, possibly leading to errors in the reconstruction. For example, the largest erroneous gap shown in the lower left of Figure 4b occurs within a 110 kbp genomic region that contains only two restriction fragments of size 40 kbp and 70 kbp, respectively. Within the same region, genomic repeats lead to a fragmentation of the de Bruijn graph resulting in a collection of short edges without any restriction site information, and one edge which contains a single restriction site. The difference in performance on the low error optical map and the experimental optical map highlights the potential benefit of developing higher resolution and more accurate mapping technologies (such as nano-coding [40]). Alternatively, additional mate-pair information (providing short-range information) along with an optical may also help resolve ambiguities in regions with few restriction sites.

### Effect of restriction enzyme choice on assembly quality

**Figure 5:**
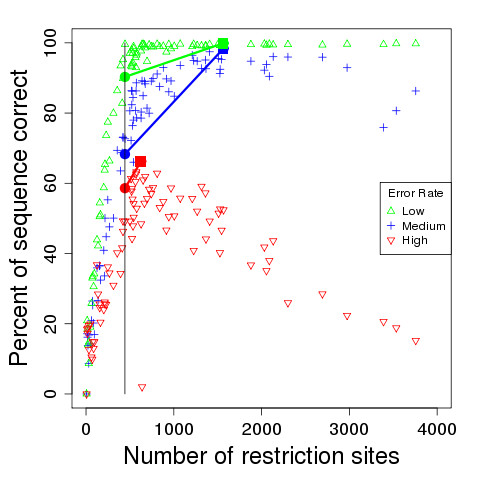
**Impact of restriction enzyme choice on assembly quality.** The choice of restriction enzymes can impact the correctness of the assembly. Each point represents the sequence correctness of an assembly of *Y. pestis* KIM when given a de Bruijn graph of k-mer size 100 and an optical map of low, medium, or high error rate. The vertical line in the picture indicates the number of restriction sites for the enzyme PvuII used to construct the experimental optical map of this genome, and the colored circles represent the correctness that can be achieved under the three error rates for the PvuII enzyme. The red, blue, and green filled squares to the right of the vertical line, indicate an improvement of between 7.7% and 30.1% in the final sequence correctness that can be achieved when choosing a better restriction enzyme in the high, medium, and low error settings, respectively.

We further examined the effect of using different restriction enzymes on the quality of the assembly that can be produced by our algorithm for the *Y. pestis* KIM genome. We generated a de Bruijn graph with k-mer size 100 for the sequence of *Y. pestis* KIM, and then computed the *in silico* map of the genome for each of 102 different restriction enzymes. We then simulated noisy optical maps by adding three different levels of noise to each *in silico* map, as described previously. In Figure 5, we plot the number of restriction sites versus the sequence correctness achieved by our algorithm using the optical map for each enzyme at the three different optical map error rates.

The vertical line in Figure 5 corresponds to the number of restriction sites for the enzyme PvuII used to construct the experimental optical map of this genome [13]. The circles drawn on the line represent the quality of the corresponding assemblies with a PvuII map under different mapping error rates. Although we are able to assemble the genome with 90.3% sequence correctness in the low error setting, the medium and high error settings only assemble with 68.3% and 58.6% sequence correctness, respectively (using the experimental map only yields 48.6% accuracy). Figure 5 illustrates that restriction enzymes that cut more frequently can yield better assemblies. A HindIII (recognition sequence A^ACGTT) optical map with 1,566 restriction sites achieves 99.8% sequence correctness in the low error setting and 98.4% in the medium error setting, as indicated by the green blue squares in Figure 5, respectively. In the high error setting, we can achieve 66.3% sequence correctness (shown as the red square) with a BSrGI (recognition sequence T^GTACA) optical map with 573 restriction sites. Over the three cases, we can improve the accuracy by between 7.7% and 30.1% by choosing an appropriate restriction enzyme.

Figure 5 also shows the dependence between the frequency with which an enzyme cuts and the quality of the resulting assembly. In the low error setting, assembly accuracy generally increases with the density of restriction sites on the optical restriction map, although this is not true for the medium and high error rates where the performance of the algorithm starts decreasing beyond a certain cut frequency. This phenomenon can be explained by the loss of more small fragments as cut frequency increases, and the increased difficulty of finding landmark edges when there are many smaller fragments of roughly the same size. In the high error setting, we note that restriction enzymes with around 500 recognition sites yield assemblies with the highest sequence correctness for *Y. pestis* KIM.

The strong dependence of the quality of assembly on the restriction enzyme used highlights the need for choosing an appropriate enzyme. Running preliminary lab experiments to digest the genome with different enzymes can be used to find an enzyme which cuts the genome at an appropriate frequency (in the case of *Y. pestis*, the ideal restriction enzyme yields an average fragment size of roughly 10 kbp). Alternatively, generating preliminary sequence data and building a corresponding de Bruijn graph, can also help estimate the cut frequency of various restriction enzymes.

### Optical maps versus mate-pairs

The use of mate-pairs to guide the assembly process was previously studied by Wetzel et al. [4] using the same genomes used in our study. A direct comparison to the full results presented previously is difficult to perform as our goal here is the reconstruction of a single contig spanning an entire chromosome, while the work of Wetzel et al. is focused on the resolution of individual repeats (and the corresponding reduction in the complexity of the assembly graph) using mate-pair information. Furthermore, mate-pairs and optical maps provide complementary types of information: mate-pairs provide local information and are most effective in the short range (as shown, e.g., in [4]) where the optical mapping resolution may be limited, while optical maps provide global information and are particularly effective in the long range (10s-100s of kbp, ranges for which mate-pair libraries are difficult to generate). To demonstrate the complementary strengths of these technologies, we highlight a couple genomes analyzed both with mate-pairs in [4] and with optical maps in our study.

First, *Rhodospirillum rubrum* ATCC 11170 (NCBI accession NC_007643) was completely and correctly resolved by AGORA in our study, but mate-pair based analyses were unable to fully resolve this genome even when trying different combinations of library sizes. We applied the mate-pair repeat resolution approach described in the work of Wetzel et al. [4] using both the tuned library mixture of sizes 477 and 6047 (see [4] for details on how the library sizes were chosen), and the ‘standard’ combinations of 2kbp + 8kbp, or 2kbp + 35kbp. Note that it is possible that some combination of two or more mate-pair libraries could have resolved this genome, as we have not exhaustively explored all possible combinations of mate-pair libraries. However, in practical terms, it is unlikely that a lab interested in solving the *Rhodospirillum* genome would attempt multiple library preparations in hopes of finding the perfect combination for this genome.

A second example is the genome of *Streptococcus agalactiae* NEM316 (NCBI accession NC_004368) which contains a 47 kbp-long plasmid-like repeat (pNEM316-1) occurring three times within the main chromosome [43]. Resolving this repeat would require mate-pairs longer than 47 kbp, which are beyond the sizes routinely generated, especially in the context of next generation sequencing technologies (fosmid libraries only extend to ~40 kbp).

### Real assembly graphs

Our results have focused on running our proof-of-principle algorithm on ideal de Bruijn graphs obtained from error-free sequencing data. The application of AGORA to data from real sequencing experiments is the object of future work and beyond the scope of this paper. However, it is natural to ask whether our algorithms can feasibly be extended to real datasets. To address this question we focused on sequencing data available for the *Yersinia pestis* KIM genome, specifically a 454 dataset (SRA accession SRX012379). We assembled these reads using Newbler [Roche] and explored the structure of the resulting contig graph (available from the 454ContigGraph.txt file produced by Newbler).

We compared the Newbler graph to the ideal de Bruijn graphs of order 100 and 500, as the average length of the 454 reads falls between these values at 438 bp. The Newbler assembly resulted in 283 contigs with an N50 size of 38,282 while the order 500 graph had 199 contigs with an N50 size of 62,865 bp and the order 100 graph had 648 contigs with an N50 size of 38,786 bp. Thus, in broad terms, the real assembly graph has similar characteristics to the perfect de Bruijn graphs in our experiments.

More relevant to our study is the question of whether landmark edges can be easily found in the Newbler graphs. The AGORA algorithm critically depends on our ability to find edges that have a unique placement along the optical map. According to this criterion, the Newbler contig graph is also roughly similar to the simulated graphs. Specifically we find 15 landmarks in the Newbler assembly, compared to 15 and 26 landmarks in the order 100 and 500 de Bruijn graphs, respectively. We also aligned the Newbler contigs using the more complex dynamic programming algorithm described in [12] and identified 22 landmarks, indicating that the use of already existing optical map alignment algorithms will be effective in extending the AGORA algorithm to real sequencing data.

## Conclusions

We have presented a computational framework that allows optical mapping data to be used during the genome assembly process. Our work demonstrates the potential of this approach in improving the assembly of bacterial genomes. With optical maps, over ¾ of our bacterial genomes were assembled with over 98% accuracy, and even the complex genome of *Y. pestis* KIM could be assembled with sequence correctness between 86.74% and 99.13%, depending on the quality of the reference optical map. Moreover, for the bacterial genomes in our test data set, in the median case we could improve on the N50 size by a factor of between 6.4 and 18.9 and reduce the number of contigs by a factor of between 6.67 and 10.74 over what could be achieved with sequence data alone.

Our initial study also allowed us to explore the effect of experimental parameters on the usefulness of mapping data. We demonstrated substantially improved quality of assembly when using high quality optical maps, highlighting the value of continued improvements in this technology (such as the nano-coding approach [40]). In addition, we showed that the choice of restriction enzyme significantly affects assembly quality, indicating the benefits of preliminary analysis to determine a suitable restriction enzyme before constructing an optical map.

The results we have shown are only a first step towards developing a map-guided genome assembler. AGORA has only been tested on error-free assembly data and will need to be adapted to handle the characteristics of assembly graphs derived from real sequencing data. The heuristics used to speed up the alignment process may not be effective in the context of a combination of realistic sequencing and mapping error profiles. A practical implementation of our approach may need to rely on a variant of the dynamic programming alignment algorithm described in [12] with additional heuristics or the use of parallel/high-performance architectures. Additionally, it may be useful to develop methods to detect regions of the assembly where multiple paths may match the optical map, and exclude those regions from the final assembly to avoid introducing errors.

Finally, a promising area of future research involves the combination of mapping and mate-pair data. These types of information offer complementary strengths – long-range structural information from optical maps, and short-range links from the mate-pair data – which can be leveraged to overcome our difficulty in resolving genomic regions that are sparsely sampled by the restriction map.

## Methods

### Optical mapping error simulations

To describe our experiments precisely, we need to formally describe the various types of noise that add error to the optical map, and how we simulate noisy optical maps for use in our experiments. In general, optical maps may have three types of errors: fragment sizing error, small fragments missing, and restriction site errors. Fragment sizing error occurs because measuring the sizes of the Rmap fragments is performed using optical techniques that associate restriction fragment mass with fluorescence intensity. Small fragments can be missing from the optical map due to desorption. Restriction site errors refer to missing or added restriction sites on the genome optical map, which can be caused by errors in the physical process or in the image processing.

To simulate optical maps for our experiments, we start by computing an *in silico* map for each genome, and then add noise to the *in silico* map to simulate fragment sizing error and small fragments lost. In our experiments, we did not extensively test restriction site errors as they are fairly rare in a finished optical occurring at around 2% of restriction sites [22]. However, we do simulate the loss of small fragments according to a small fragment threshold μ ≥ 0, as well as fragment sizing error according to two parameters α ≥ 1 and β ≥ 0. Using fluorescence intensity to estimate restriction fragment length leads to an error proportional to the length of the fragment, which we characterize with a multiplicative error parameter (α). Smaller fragments have different factors contributing to their error profile, however, which we characterize with an additive error parameter (β). (For more details on optical mapping error models, see [10,44].)

Given an *in silico* map and the parameters described above, we start by deterministically removing all fragments of size less than μ, which is the worst case for small fragment loss, as some small fragments are retained in practice.

Next to simulate fragment sizing error for parameters α, β, and μ, we add a random amount of noise to the remaining *in silico* fragments, so that an *in silico* fragment of size S may produce an optical map fragment of size between a lower bound L = max(S/α – β, μ) and upper bound U = αS + β. For each fragment of size S, we substitute a fragment of length S + ϵ, where ϵ is Gaussian noise with mean 0 and standard deviation (U-L)/4. If S + ϵ < L or S + ϵ > U, then we substitute L or U, respectively.

The three parameters α, β, and μ are then used to model the three levels of noise used for our experiments. In the **low error** setting, we set α = 1.01, β = 100 bp, and μ = 0 (which did not allow for any small fragments to be lost). In the **medium error** setting, we set α = 1.05, β = 1000 bp, and μ = 1000 bp. In the **high error** setting, we set α = 1.10, β = 2000 bp, and μ = 2000 bp. The high error setting has bounds on the maximum sizing error and maximum size of small fragments lost, corresponding to the values observed in the published optical map of the *Y. pestis* KIM genome [13]: up to 10% (α = 1.10) multiplicative and 2000 bp additive fragment sizing error; in addition, small fragments up to size 2000 bp were lost (and no restriction site errors were observed). The low error rate setting, which did not allow for small fragments to be lost (μ = 0), may eventually be achievable using the nano-coding system currently being developed [40]. Note that our error simulation does not fully capture all the factors that affect the quality of experimental optical maps, which causes differences between the performance of our algorithm when applied to simulated and experimental optical maps. We opted for a simplified model in order to enable the detailed simulations described in our paper; however we plan to further investigate more realistic error models in future work.

### AGORA algorithm

High level pseudocode illustrating the basic idea of the AGORA algorithm is provided below. A more detailed explanation describing additional improvements to the basic algorithm is given in the following sections. The source code for AGORA is provided as Additional file 3, along with code needed to run our experiments. AGORA takes as input two data structures: OpMap – an ordered list of fragment sizes representing the optical map; and Edges – a list of de Bruijn graph edges with their corresponding sequences.

### AGORA(OpMap, Edges)

Set *LandmarkEdges* = FindLandmarkEdges(*Opmap*, *Edges*)

Sort *LandmarkEdges* in order of their position on the optical map

For circular genomes, add a copy of the first landmark edge to the end of *LandmarkEdges*

Set *CurrentEdge* to be NULL_POINTER

Set *CurrentPath* to be the empty path

Push the first edge of *LandmarkEdges* onto the top of *EdgeStack*, a stack of edges to be explored in the DFS

**For each pair of consecutive edges (E**_**1**_**, E**_**2**_**) in*LandmarkEdges***

// Perform a depth first search from *E*_*1*_ until *E*_*2*_ is

// reached with a path matching the optical map

**While (*CurrentEdge*!=*E***_***2***_**)**

*CurrentEdge* = Pop top element of *EdgeStack*

**If** (*CurrentEdge* == NULL_POINTER) **then**

Backtrack by removing last edge from *CurrentPath*

Else

**If** the *in silico* map of *CurrentPath* + *CurrentEdge* matches the optical map **then**

*CurrentPath* = *CurrentPath* + *CurrentEdge*

Push NULL_POINTER onto *EdgeStack* for backtracking

Push each edge outgoing from the end of *CurrentEdge* onto *EdgeStack*

EndIf

EndIf

EndWhile

EndFor

EndProgram

#### Finding landmark edges

The first step of AGORA computes landmark edges, which are edges in the graph that have a unique placement within the reference optical map. These landmark edges are found by computing an *in silico* map from the sequence of each edge, and checking if the *in silico* map can be placed at exactly one location by attempting to align the *in silico* map starting from each fragment in the genome optical map. We implemented a simple greedy algorithm to align an *in silico* map to an optical map alignment, although a more precise dynamic programming algorithm was described previously in [12]. We used a heuristic approach instead of the more accurate alignment algorithm in order to speed up landmark computation. The dynamic programming algorithm has run-time proportional to the fourth-power of the number of fragments being aligned.

Our greedy alignment algorithm simply compares *in silico* fragments to optical map fragments in order, allowing for a size mismatch within the bounds specified by the (α, β) parameters, and allowing fragments of size smaller than μ to be missing. (In the experiments, the α, β, and μ parameters are set according to the values used to simulate the optical maps, or in the case of the *Y. Pestis* KIM experimental optical map, we set α = 1.10, β = 2,000 bp, and μ = 2,000 bp).

The greedy algorithm does not allow restriction site errors in the optical map alignment when determining landmark edges. Although restriction site errors may occur in practice, we do not allow restriction site errors when determining landmark edges to limit ambiguous placements and help ensure that all our landmark edges have correct placements on the genome optical map. This greedy alignment algorithm has a linear run-time, which was made even more efficient by saving the alignments of previous edges in the path and progressively aligning new edges added to the path during the depth-first search process.

In case no landmark edges can be found, we search for a *landmark pair* – a pair of consecutive edges whose combined sequence and corresponding *in silico* map has exactly one valid alignment to a single location in the optical map. An alignment is valid if the sizes of consecutive restriction fragments within the optical map and the *in silico* map are approximately matched, modulo sizing errors and potential loss of small fragments. If a landmark pair is found, we use it to start our depth first search instead (not shown in the pseudocode).

#### Landmark to landmark path search

After determining landmark edges, we search for paths connecting consecutive landmark edges, starting with the landmark with the earliest placement within the reference optical map. (Although our bacterial genomes are circular, the experimental and simulated optical maps are provided to the algorithm as an ordered list of fragment sizes, which is used to define the earliest placement in the optical map.) After the search reaches the last landmark edge, we search for a path connecting the last landmark edge to first landmark edge to finish the path, since we were assembling circular bacterial genomes. In case only one landmark edge can be found, we search for a path from the one landmark edge back to itself. If no landmark edges can be found, but a landmark pair can be found, then we attempt to find a path from the landmark pair back to itself (otherwise we return with no path found).

To find a path between pairs of consecutive landmark edges (or from a landmark edge or landmark pair back to itself), we rely on a depth-first search algorithm, pruning the search space according to the following criteria. An edge can only be used to extend the current depth-first search path if the *in silico* map of the path matches the optical map, and three additional conditions hold (not shown in the psuedocode):

1. the edge is not currently used in the current path (if multiple edges have been collapsed into a single edge, we ensure an edge is not used more times than its multiplicity);

2. the *in silico* map alignment of CurrentPath to the optical map does not extend past the first restriction site of the alignment of the next consecutive landmark edge;

3. the edge currently being added to the depth first search has not been explored more than 500 times previously, while being aligned at the same optical map location (this step avoids repeatedly exploring very many similar paths within a highly complex region of the genome).

In AGORA’s depth first search implementation, we explore edges in decreasing order of length (exploring edges with the longest sequence length first). Their longer length often makes those edges the easiest to accurately place along the optical map.

#### Modifications to improve efficiency

In preliminary tests, we found the depth first search can incorrectly traverse an edge in the path between two landmark edges early in the search process, which prevents the correct path from being found between subsequent landmark edges without substantial backtracking. When no path can be found between two consecutive landmark edges without backtracking through previously explored landmark edges, we simply ‘restart’ the search from the current landmark edge with the algorithm assuming that no edges have been traversed so far. The search should succeed this second time, since edges which may have been incorrectly used in the prior path are now available to be explored again.

Additionally, if the algorithm fails to find a path between landmarks within a preset amount of time (we used one minute in our simulations), we simply skip to the next landmark without attempting to reconstruct the region between the landmarks. In our experiments, we did not have to use this procedure often, but the additional check was useful for a small set of complex genomes to ensure completion within a reasonable amount of time.

It is important to note that the various heuristics described above, while dramatically improving the performance of our algorithm, lead to potential errors in the reconstruction, especially when using lower quality mapping data. We plan to explore the tradeoff between accuracy and performance in future work.

### Edge and sequence correctness metrics for measuring assembly quality

Before describing the edge correctness and sequence correctness metrics more precisely, it is important to note a significant difference between AGORA and typical genome assembly algorithms. Our algorithm seeks to construct a single contig representing the full genome of the organism being assembled, while accepting some errors, in contrast to most assemblers, which break the assembly into separate contigs to avoid assembly errors. As a result, traditional metrics of assembly quality do not directly apply in our case, and thus we propose the alternative metrics described below. In brief, we attempt to compare the traversal of the de Bruijn graph chosen by AGORA to the true traversal representing the correct genome sequence. We measure the concordance between these two paths in terms of both number of concordant edges and similarity between the reconstructed sequences. We term the two measures edge correctness and sequence correctness, respectively.

To compute the *edge correctness* measure, we start by matching the path found by AGORA to the correct path through the graph using a longest common subsequence algorithm. The edges not aligned by this algorithm correspond to errors in our reconstruction. The edge correctness metric overestimates the amount of error in the reconstruction. In many cases the errors correspond to short edges and thus do not significantly affect the overall correctness of the reconstructed sequence.

To account for this issue, we also computed a metric which we called *sequence correctness*, which weights the edge correctness metric by the actual length of the edges. More precisely, we implement a weighted longest common subsequence algorithm to identify the ‘heaviest’ set of edges that match the correct path in the correct order. We then sum the length of these edges and divide by the total genome sequence length to obtain our sequence correctness metric.

One last caveat we should mention is that if we ever find two different edges between the same nodes in the de Bruijn graph with greater than 99% sequence similarity, then we treat them as if they were the same edge, as long as the sequence differences do not cause any change to their restriction sites. This procedure of collapsing similar edges is known as “bubble collapsing” and is useful for handling nearly equivalent edges within the de Bruijn graph. Such edges are impossible to disambiguate through optical mapping, and we ignore any errors we might make by swapping the order in which they are traversed. Note that even if we were to measure the additional differences in the sequence produced by AGORA that occur due to bubble collapsing which are ignored in the sequence correctness score, the overall decrease in the percent of sequence matching the true genome is at most 1%, since we only collapse bubbles that are at least 99% identical.

## Competing interests

The authors declare that they have no competing interests.

## Authors’ contributions

MP and HL designed the main idea for the algorithm with advice and suggestions from DCS and SG. HL implemented and ran the experiments with help from LM. SZ provided the optical map data. JW provided the de Bruijn graphs and helped compare the optical map results with the previous mate-pair experiments. MP and HL wrote the manuscript with edits and suggestions from DCS, SG, LM, and JW. All authors have read and approved the manuscript.

## Supplementary Material

Additional file 1 **Per-genome results of assembling each genome.** An excel spreadsheet detailing the results of assembling 369 bacterial genomes with AGORA, given optical maps simulated with three different error rates. Click here for file

Additional file 2 **Figure showing the impact of starting N50 size of the de Bruijn graph on sequence correctness.** A plot showing the sequence correctness of 369 bacterial genome assemblies by AGORA versus the starting N50 size of their de Bruijn graphs, under three different optical map error rates. Genomes with starting N50 size greater than 50 kbp are generally assembled with higher than 98% correctness over all three error rates, while the results are mixed for genomes with lower starting N50 size. Click here for file

Additional file 3 **AGORA source code.** A zip file containing the code used to generate the results presented in our paper. Click here for file
