# Supplementary figures and images for: AGORA: Assembly Guided by Optical Restriction Alignment
Source: BMC Bioinformatics. 2012 Aug 2;13:189. doi: 10.1186/1471-2105-13-189 (PMC3431216; doi:10.1186/1471-2105-13-189)

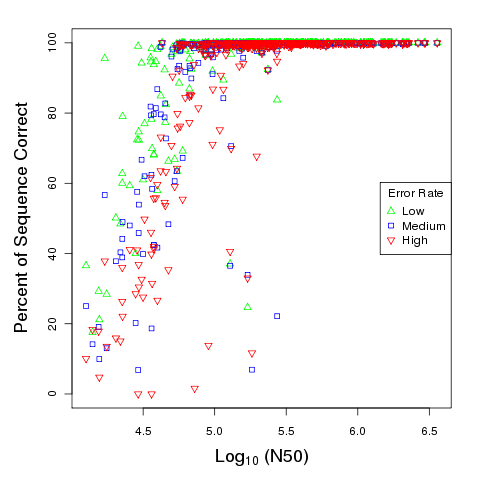

Supplement: Additional file 2 — Figure showing the impact of starting N50 size of the de Bruijn graph on sequence correctness. A plot showing the sequence correctness of 369 bacterial genome assemblies by AGORA versus the starting N50 size of their de Bruijn graphs, under three different optical map error rates. Genomes with starting N50 size greater than 50 kbp are generally assembled with higher than 98% correctness over all three error rates, while the results are mixed for genomes with lower starting N50 size. [file 1471-2105-13-189-S2.png]
